# Characterization and Phytotoxicity Assessment of Essential Oils from Plant Byproducts

**DOI:** 10.3390/molecules24162941

**Published:** 2019-08-14

**Authors:** Antonella Smeriglio, Domenico Trombetta, Laura Cornara, Marco Valussi, Vincenzo De Feo, Lucia Caputo

**Affiliations:** 1 Department of Chemical, Biological, Pharmaceutical and Environmental Sciences, University of Messina, Viale Giovanni Palatucci, 98168 Messina, Italy; 2 Department for the Earth, Environment and Life Sciences, University of Genova, Corso Europa 26, 16132 Genova, Italy; 3 European Herbal & Traditional Medicine Practitioners Association (EHTPA), Office 3, 47 St Giles Street, Norwich NR2 1JR, UK; 4 Department of Pharmacy, University of Salerno, Via Giovanni Paolo II 132, 84084 Fisciano, Salerno, Italy

**Keywords:** *Zingiber officinale* Roscoe, *Pistacia vera* L., *Cannabis sativa* L., *Cupressus sempervirens* L., essential oil, phytotoxicity

## Abstract

The present work describes the chemical characterization and the phytotoxicity assessment of essential oils (EOs) obtained from spent materials or pruning waste of four plant species: *Zingiber officinale* Roscoe used in the juicing industry, *Pistacia vera* L. var. *Bronte* used in the food industry, discarded material of industrial hemp (*Cannabis sativa* L. var. *Futura 75*), and pruning waste from *Cupressus sempervirens* L. The phytochemical profile of the EOs was evaluated by gas chromatographic flame ionization detection (GC-FID) and GC-MS analyses, which highlighted the presence of several compounds with a wide range of biological activities. Among them, application possibilities in agriculture were evaluated by studying the phytotoxic activity *in vitro* against germination and initial radical growth of several seeds such as *Raphanus sativus* L., *Lepidium sativum* L., *Lactuca sativa* L., *Solanum lycopersicum* L., *Lolium multiflorum* Lam., and *Portulaca oleracea* L.

## 1. Introduction

Modern agriculture uses many synthetic herbicides to manage weeds, and about 2.5 million tons of pesticides are used every year [1,2]. Over the years, the excessive use of synthetic herbicides and pesticides has harmed the environment and human health. Considering this, recently, much has been invested in the study of alternative strategies that can lead to the development of biodegradable and nontoxic products [3]. This is because many old herbicides have been withdrawn from the market for safety issues and there has been rapid evolution of resistance to new synthetic herbicides [4]. Moreover, the development of insecticide resistance has resulted in the loss of food [5]. 

Allelopathy is a biological phenomenon that affects the growth and development of plants using secondary metabolites produced in nature [6]. These latter compounds are also able to defend plants against phytopathogenic bacteria and/or fungi, plant-feeding insects, and herbivores [5,7].

Among the most investigated natural sources of secondary metabolites, essential oils (EOs) play a pivotal role [8]. 

In the last decade, many EOs have been studied for their phytotoxic and pesticidal properties, with terpenes being the class of bioactive compounds to which these biological activities are mainly attributable, although with generally higher concentrations needed to obtain phytotoxic effects to be used as insecticides or fungicides [9,10,11,12].

Indeed, it has been shown that many highly phytotoxic allelochemicals are derived from the terpenoid biosynthetic pathway [3].

Recently, considerable attention from the scientific community has been paid to the recovery of waste processing products from the agricultural and food industry. Indeed, it is well known that such waste products are an economic burden for processing companies due to the transport and disposal costs as special waste [13]. In other cases, biomass and byproducts are sources of compounds with technological and nutritional properties [14]. Moreover, the biomass of plant species subjected to pruning is rich in EOs.

In light of this, the aim of this study was to evaluate the phytochemical profile and the *in vitro* phytotoxic effects of the EOs isolated from *Zingiber officinale* Roscoe (Zingiberaceae, ginger), *Pistacia vera* L (Anacardiaceae, pistachio)., *Cannabis sativa* L. var. *Futura 75* (Cannabaceae, hemp), and *Cupressus sempervirens* L. (Cupressaceae, cypress) byproducts on the germination and initial radical elongation of several seeds such as *Raphanus sativus* L. (radish), *Lepidium sativum* L. (garden cress), *Lactuca sativa* L. (lettuce), *Solanum lycopersicum*, L. (tomato), *Lolium multiflorum* Lam. (ryegrass), and *Portulaca oleracea* L. (purslane).

## 2. Results

### 2.1. Micromorphological Analysis

**Figure 1:**
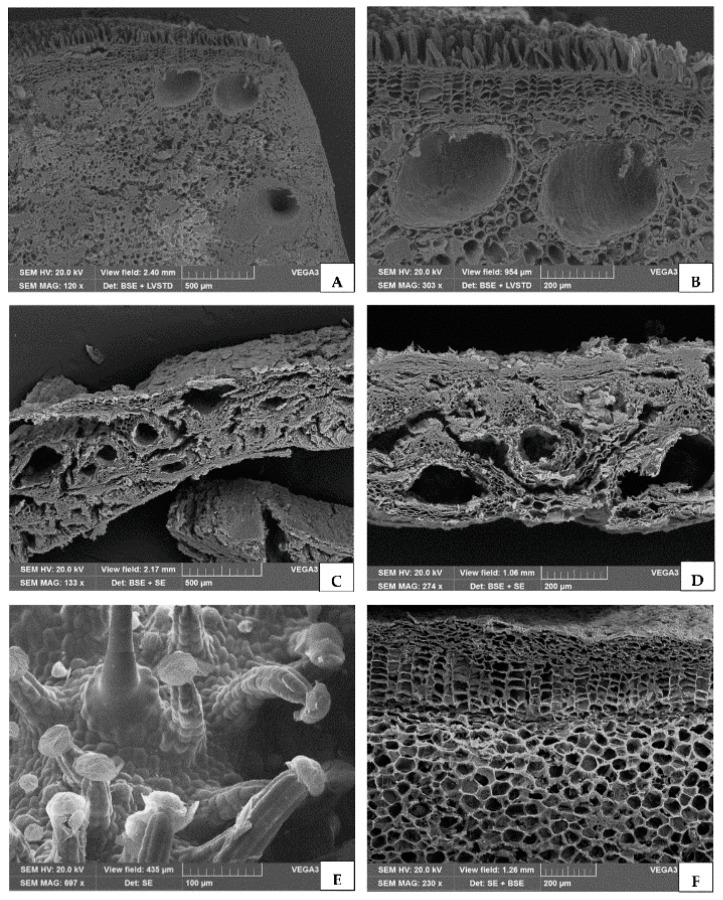
SEM micrographs of different plant tissues used for EO isolation. (**A**,**B**) Cross section of a cone scale from *Cupressus sempervirens* L., showing several resin ducts at different magnifications. (**C**,**D**) Particular of the cross section of the mesocarp from the *Pistacia vera* L. var. *Bronte* hull, showing many resin ducts. (**E**) Inflorescences of *Cannabis sativa* L. var. *Futura 75* showing long, multicellular, stalked glandular trichomes with large heads where resin is produced and stored. (**F**) Section of *Zingiber officinale* Roscoe rhizome containing oil globules in secretory cells scattered in the parenchyma.

Samples from various species characterized by different kinds of secretory tissues, in which EO is synthesized and accumulated, were investigated. In female cones of *C. sempervirens*, the cross section of a cone scale showed several schizogenous resin ducts (Figure 1A,B). The hull of *P. vera* var. *Bronte* showed many resin ducts in the mesocarp, forming large schizogenous cavities where EO is synthesized (Figure 1C,D). Inflorescences of *C. sativa* var. *Futura 75* showed long, multicellular, stalked glandular trichomes with large heads, where resin is produced and stored, together with a few small glandular trichomes with a bicellular head and a unicellular stalk (Figure 1E). In the rhizome of *Z. officinale*, a well-marked endodermis separates the cortex from vascular tissue, while oil globules are present in secretory cells scattered in the parenchyma (Figure 1F).

### 2.2. Essential Oil Yields and Chemical Composition 

Hydrodistillation of the aerial parts of *C. sativa*, hulls of *P. vera*, and cones of *C. sempervirens* furnished oils in 0.2%, 0.3%, and 0.6% yields on a dry mass basis, respectively, while hydrodistillation of the fresh rhizomes of *Z. officinale* furnished oil in a 0.7% yield.

The compositions of the EOs, with retention indices and area percentages for each compound, are given in Table 1, Table 2, Table 3 and Table 4.

Seventy-nine compounds were detected in the *C. sativa* var. *Futura 75* EO (Table 1). 

Sesquiterpenes represent the most abundant class (52.26%), followed by monoterpenes (40.51%), oxygenated sesquiterpenes (4.87%), cannabinoids (1.24%), and oxygenated monoterpenes (0.52%). Major sesquiterpenes include α-caryophillene (21.68%), β-caryophillene (9.86%), caryophillene oxide (3.83%), α-bergamotene (3.22%), selina-3,7(11)diene (2.54%), and δ-Guaiene (2.16%). The most abundant monoterpenes are α-terpinolene (9.35%), β-myrcene (9.32%), α-(+)-pinene (7.82%), trans-β-ocimene (4.62%), (1S)-(−)-β-pinene (3.73%), and D-limonene (2.92%). Among cannabinoids, cannabidiol is the most representative (1.17%).

Into the EO of *P. vera* L. var. *Bronte* hull, 40 compounds were detected (Table 2), belonging mainly to the class of monoterpene hydrocarbons (86.20%), and oxygenated monoterpenes (11.37%) and sequiterpenes (0.21%) are less represented. 

Among monoterpenes, the most abundant compounds are 4-carene (32.03%), α-pinene (22.65%), D-limonene (8.50%), δ-3-carene (7.98%), α-terpineol (3.99%), camphene (3.88%), β-myrcene (2.43%), bornyl acetate (2.37%), and α-terpinene (2.33%).

*Z. officinale* EO showed a chemical composition similar to hemp EO regarding the relative abundance of terpene classes. In fact, among the 66 compounds identified, the most abundant ones belong to sesquiterpene hydrocarbons (60.57%), followed by monoterpenes (22.72%), oxygenated monoterpenes (11.73%), and oxygenated sesquiterpenes (1.71%). Major sesquiterpenes include α-zingiberene (15.92%), γ-amorphene (11.55%), γ-patchoulene (8.77%), β-acoradiene (5.72%), α-acoradiene (5.27%), and *allo*-aromadendrene (2.27%). Among monoterpenes, the most abundant are eucalyptol (10.32%), santolina triene (6.29%), isobornyl formate (4.89), and α-pinene (2.47%) (Table 3).

Finally, *C. sempervirens* EO showed the presence of 48 compounds belonging predominantly to monoterpene hydrocarbons (81.34%). Sesquiterpene hydrocarbons (13.30%), oxygenated monoterpenes (3.96%), and oxygenated sesquiterpenes (0.94%) were in less amounts (Table 4). 

The most abundant monoterpenes are α-pinene (43.25%), *p*-mentha-1(7),8-diene (16.47%), (E)-β-ocimene (4.49%), *p*-mentha-2-4(8)-diene (3.76%), sabinene (3.24%), *allo*-ocimene (2.61%), and α-terpinyl acetate (2.25%). α-Cedrene (3.97%) and α-gurjunene (2.01%) predominated in sesquiterpenes.

The pistachio hull EO showed the highest content of monoterpenes, followed by cypress EO, while the ginger EO showed the highest content of sesquiterpenes, followed by hemp EO.

### 2.3. Phytotoxic Activity

**Figure 2:**
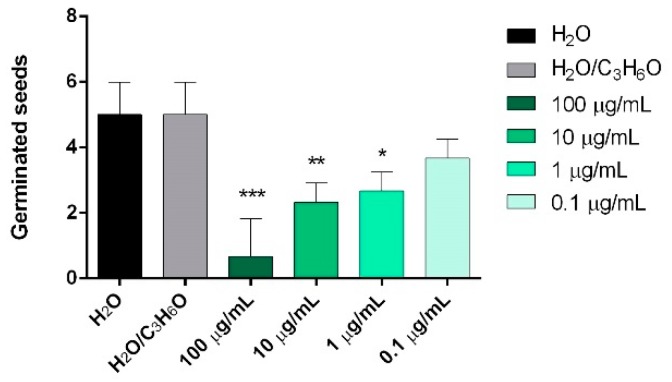
Phytotoxic activity of *P. vera* EO against germination of *Portulaca oleracea* L., 120 h after sowing. Results are the mean ± standard deviation (*n* = 3) of three independent experiments. * *p* < 0.05, ** *p* < 0.01, *** *p* < 0.001 compared to control (ANOVA followed by Dunnett’s multiple comparison test).

In order to evaluate the in vitro phytotoxic activity of the selected EOs, six seeds (radish, garden cress, lettuce, tomato, ryegrass, and purslane) were used, estimating their germination and radical elongation. Only *P. vera* EO showed activity against the germination of *P. oleracea* seeds after a treatment with 100, 10, and 1 μg/mL (Figure 2). 

**Figure 3:**
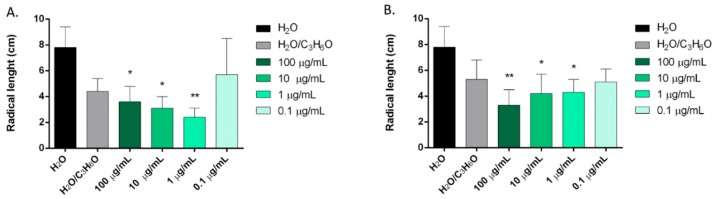
Phytotoxic activity of *C. sempervirens* (**A**) and *Z. officinale* (**B**) EOs against radical elongation of *Lepidium sativum* L., 120 h after sowing. Results are the mean ± standard deviation (*n* = 3) of three independent experiments. * *p* < 0.05, ** *p* < 0.01 compared to control (ANOVA followed by Dunnett’s multiple comparison test).

**Figure 4:**
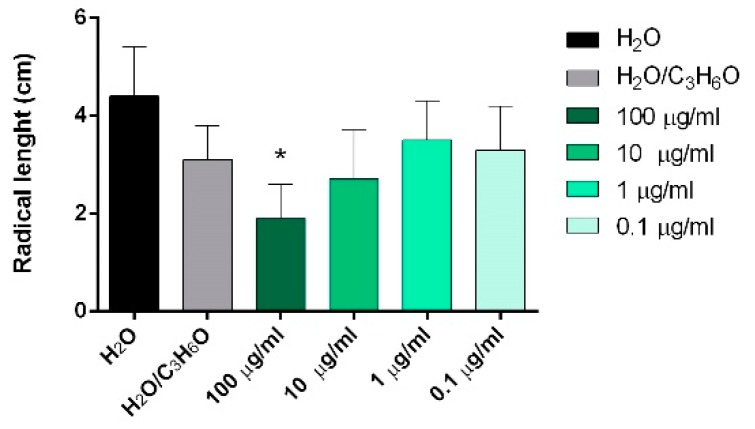
Phytotoxic activity of *Z. officinale* EO against radical elongation of *Lolium multiflorum* Lam.,120 h after sowing. Results are the mean ± standard deviation (*n* = 3) of three independent experiments. * *p* < 0.05, compared to control (ANOVA followed by Dunnett’s multiple comparison test).

**Figure 5:**
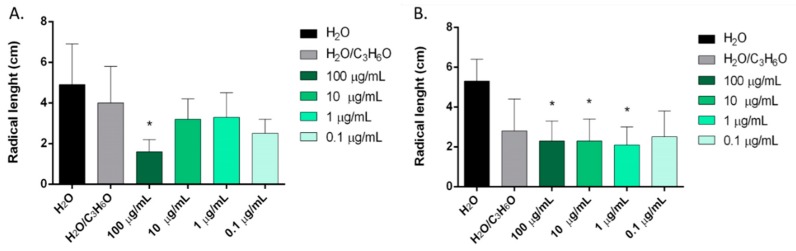
Phytotoxic activity of *P. vera* (**A**) and *C. sativa* (**B**) EOs against radical elongation of *Raphanus sativus* L., 120 h after sowing. Results are the mean ± standard deviation (*n* = 3) of three independent experiments. * *p* < 0.05, compared to control (ANOVA followed by Dunnett’s multiple comparison test).

*C. sempervirens*, *Z. officinale*, *C. sativa*, and *P. vera* EOs showed, in different ways, statistically significant activity against initial radical elongation on *L. sativum*, *L. multiflorum*, and *R. sativus* seeds (Figure 3, Figure 4 and Figure 5).

*C. sempervirens* and *Z. officinale* EOs were able to inhibit the radical elongation of *L. sativum* at concentrations of 100, 10, and 1 μg/mL (Figure 3). 

Moreover, *Z. officinale* EO was the only one that also showed phytotoxic activity against the radical elongation of *L. multiflorum* seeds at the highest concentration tested (100 μg/mL) (Figure 4).

*P. vera* hull and *C. sativa* EOs were active against the radical elongation of *R. sativus* (Figure 5). *P. vera* EO inhibited the radical elongation at the highest concentration used (100 μg/mL), and *C. sativa* var. *Futura 75* EO inhibited radical elongation at 100, 10, and 1 μg/mL.

## 3. Discussion

In this study, the chemical composition and possible phytotoxic activity of EOs obtained from different plant sources were investigated. In particular, EOs from the spent materials of *Z. officinale*, used in the juicing industry; discarded material of *P. vera*, used in in the food industry; discarded material of industrial hemp (*C. sativa* var. *Futura 75*); and pruning waste from the tree *C. sempervirens* were obtained. All these materials are generally considered to be byproducts or waste products; however, they can be a rich source of EOs with a wide range of applications. 

Secretory tissues occurring in most vascular plants differ not only in structure and localization but also in terms of the secreted material. Different plant species synthesize lipophilic substances, such as EOs and resin, which have been used by humans throughout the ages for many purposes and are still of interest for environmental, agricultural, food, and medical applications. Secretory tissues may consist of single cells or hydathodes (e.g., in the *Zingiber* rhizome) or small to very large groups of cells. Glandular trichomes are located on the plant surface, such as in the female inflorescence of *C. sativa*, while secretory ducts are located inside the plant organs, as occurs in the resin ducts of the *C. semprevirens* cones and the *P. vera* hull. 

There are few studies in the available literature on the chemical composition of *C. sativa* var. *Futura 75*. The EO analyzed in the present study showed a similar composition with respect to an EO isolated from hemp leaves by Benelli et al., with sesquiterpene hydrocarbons as the main constituents (52.5%) [15]. However, Nissen et al. showed a completely different phytochemical profile, with monoterpenes as the most abundant components [16].

The phytochemical profile of the EO from *P. vera* var. *Bronte* hull was superimposable with that which was previously analyzed; this is essentially due to the collection time and place, which were the same as previously reported [17]. However, the chemical composition of the EO reported in this study disagrees with results reported for *P. vera* EO belonging to other varieties and from different countries. *P. vera* EO var. *Mateur* from Tunisia is rich in α-pinene (42.5%) and terpinolene (32.2%), while the latter compound is totally absent in the sample here investigated [18]. Hashemi-Moghaddam et al. reported α-pinene (31.8%), α-terpinolene (20.3%), and myrcene (12.2%) as the main components of the EO obtained from *P. vera* var. *Shahpasand* hull cultivated in Iran [19]. 

*Z. officinale* EO was richest in sesquiterpenes, with a higher concentration of α-zingiberene than those reported in previous studies [20,21]. However, Lagha et al. already reported the most abundant presence of sesquiterpenes and α-zingiberene as the main compound in *Z. officinale* EO from France [22].

Few studies have analyzed the chemical composition of *C. sempervirens* cone EO. Our results are in accordance with Milos et al. [23] and Tumen et al. [24], who reported α-pinene as the main constituent of *C. sempervirens* cone EO, with percentages of 69.9% and 66.7%, respectively. Selim et al. also showed this as the main constituent in the EO from the aerial parts of *C. sempervirens* oil [25].

The possible phytotoxic effects of the EOs against germination and initial radical elongation of *R. sativus* L., *L. sativum* L., *L. sativa* L., *S. lycopersicum*, L., *L. multiflorum* Lam., and *P. oleracea* L. were evaluated. Few studies have been carried out to investigate the potential phytotoxicity of EOs against these six selected species. 

In the present work, none of the EOs inhibited germination or radical elongation of *S. lycopersicum*. Nevertheless, Rolli et al. showed that *C. sativa*, *Z. officinale*, and *C. sempervirens* EOs are able to inhibit *S. lycopersicum* root length, with percentages of 47.9%, 73.8%, and 0.6%, respectively [26]. 

These differences are attributable to the different phytochemical profiles of the EOs investigated in the present work, which certainly influenced their phytotoxic activity [27]. Indeed, *C. sativa* EO showed a completely different phytochemical profile with respect to that reported in the previous work, with sesquiterpenes as the most abundant compounds with respect to monoterpenes and, in any case, a very low presence of oxygenated metabolites. *C. sempervirens* EO showed a similar phytochemical distribution, with monoterpenes as the most abundant compounds with respect to sesquiterpenes but, also in this case, with a low presence of oxygenated compounds. Finally, *Z. officinale* EO, even regarding the sesquiterpenes and monoterpenes ratios, showed a total inversion in the oxygenated sesquiterpenes content, which in the previous work represented about 99.70% of the total sesquiterpenes, compared with that investigated in the present work, in which they represent only 2.75%. In light of this, it is possible to hypothesize that the phytotoxic activity detected by Rolli et al. [26] against *S. lycopersicum* was mainly due to the presence of oxygenated metabolites and, in particular, to the oxygenated sesquiterpenes given the highlighted order of potency: *Z. officinale* > *C. sativa* > *C. sempervirens*.

The phytotoxic effect of an EO obtained by female inflorescences of another fiber hemp cultivar (Bialobrzeskie) was observed also against germination of other weeds and crops with redroot pigweed and rye brome, which resulted the most susceptible plant species. On the contrary, oilseed rape and oats have shown the most resistance [28].

Although in this study *Z. officinale* and *C. sempevirens* EOs showed radical elongation inhibition of *L. sativum* seeds, in literature, there are no other data on the phytotoxic activity of *Z. officinale* EO on this plant species and only one study showing a strong inhibitory effect of *C. sempervirens* aqueous extract on seed germination of lettuce, radish, and tomato [29,30].

*C. sativa and P. vera* EOs were active against radical elongation of *R. sativus.* Moreover, *Z. officinale* and *P. vera* EOs were active against the two weed species *L. multiflorum* and *P. oleracea*. 

These results agree with Ismail et al., who showed that *P. vera* and *P. terebinthus* L. EOs strongly inhibited the germination and seedling growth of *Sinapis arvensis* L., *Trifolium campestre* Schreb., *Lolium rigidium* Gaudin, and *Phalaris canariensis* L. in a dose-dependent manner. No previous data were present on the possible phytotoxic activity of *C. sativa* EO on similar weed species [31].

However, recently, the allopathic effect of water extracts of fiber hemp on the germination energy and rate of monocot (spring wheat and winter rye) and dicot (yellow lupine and winter rape) crop species were investigated. Hemp extract decreased the germination rate in particular of monocot plants, although all four species investigated produced shorter roots at the highest concentration tested [32]. 

EOs are reported in literature for their phytotoxic activity, acting as inhibitors of both seed germination and radical elongation, with different potencies [3,33]. Monoterpenes and, in particular, oxygenated compounds seem to be responsible for such activity, above all when they are ketones, alcohols, aldehydes, and phenols [34,35].

However, it has been demonstrated that herbicidal activity as well as antimicrobial and insecticide activity is due to the synergy of major constituents of EOs with less phytotoxic components [36].

An advantage of the use of EOs as botanical pesticides with respect to synthetic ones, other than the already well-studied and endorsed toxic effects on the human health of the latter, is the development of growing resistance. The use of botanical pesticides and, in particular, of EOs could solve this problem because it has been demonstrated that complex mixtures rarely, or at least more slowly, cause resistance phenomena. The disadvantages of EOs are certainly the volatility and the high cost. It has been demonstrated that post-application temperature influences the insecticidal activity of EOs [37]. Also, if to date no data were available about the possible repercussions of temperature on the anti-germination activity of EOs, since they are very volatile compounds, an indirect correlation could be hypothesized.

In light of this, the results of the present research open new perspectives in the reutilization of byproducts of aromatic plants. In fact, this waste material can be used for some agricultural practices, with evident economic and environmental advantages. In particular, mulching with aromatic plant byproducts has been proposed as an effective method in organic agriculture [38,39]. 

## 4. Materials and Methods

### 4.1. Chemicals

C7–C40 saturated alkane standard mix and Na_2_SO_4_ were purchased from Sigma-Aldrich (Milan, Italy). Terpene standards (≥98%) were purchased from Extrasynthese (Genay, France). GC-grade dichloromethane was purchased from Merck (Darmstadt, Germany).

### 4.2. Plant Material and Isolation of Essential Oil 

Spent materials or pruning waste from *Z. officinale*, *C. sativa* var. *Futura 75*, and *C. sempervirens* were obtained from FX Laboratorio Benessere s.r.l., Vicenza (Italy). *P. vera* var. *Bronte* hull was obtained from a local farmer in Bronte (Catania, Italy). Plant materials were subjected to steam distillation until no significant increase in the volume of the collected EO was observed (3 h). EOs were dried on Na_2_SO_4_ and stored in a dark-sealed vial with nitrogen headspace until analysis.

### 4.3. Micromorphological Analysis

Small pieces of each sample, approximately 1 cm^2^, were sectioned with a razor blade and fixed overnight at 4 °C in FineFIX working solution (Milestone s.r.l., Bergamo, Italy) with 70% ethanol, according to Chieco et al. [40]. The specimens were then dehydrated through a graded series of ethanol (80%, 90%, 95%, and 100%) and finally in CO_2_ by a critical point dryer apparatus (K850 CPD 2M Strumenti S.r.l., Roma, Italy). Dried samples were mounted on stubs, coated with 10 nm of gold, and observed with a Vega3 Tescan LMU scanning electron microscope (SEM) (Tescan USA Inc., Warrendale, PA, USA) at an accelerating voltage of 20 kV.

### 4.4. Gas Chromatographic Flame Ionization Detection (GC-FID) and GC-MS Analysis

GC analysis was performed on an Agilent gas chromatograph, model 7890A, equipped with a flame ionization detector (Agilent Technologies Santa Clara, CA, USA). An HP-5MS capillary column (30 mm, 0.25 mm coated with 5% phenyl methyl silicone, 95% dimethyl polysiloxane, 0.25 μm film thickness) and helium as the carrier gas (1 mL/min) were used. One microliter of 10% essential oil/CH_2_Cl_2_ *v*/*v* was injected by split mode (50:1). The injector and detector temperature were 250 °C and 280 °C, respectively. The following elution program was used: 60 °C for 6 min, increased to 270 °C at 3 °C/min, and held at 270 °C for 4 min. Percentages of compounds were determined from their peak areas in the GC-FID profiles. GC-MS analysis was carried out on the above instrument, coupled with an Agilent 5975C mass detector with the same column and the same operative conditions used for the analytical GC. We adjusted the ionization voltage to 70 eV, the electron multiplier to 900 V, and the ion source temperature to 230 °C. 

### 4.5. Identification of the Essential Oil Components

Mass spectra data were acquired in scan mode (*m*/*z* range of 45–450 amu). Detected compounds were identified based on the following parameters: GC retention index (relative to C7–C40 n-alkanes on the HP-5MS column), values reported in the literature [41], matching of mass spectral data with those of the MS library (NIST 08) [42], comparison of MS fragmentation patterns with those reported in the literature, and co-injection with commercially available terpene standards.

### 4.6. Phytotoxic Activity

The phytotoxic activity was evaluated on the germination and radical elongation of six different plant species: *R. sativus* L. (radish), *L. sativa* L. (lettuce), *L. sativum* L. (garden cress), *S. lycopersicum* L. (tomato), *L. multiflorum* Lam. (ryegrass), and *P. oleracea* L. (purslane). These seeds are usually used in assays of phytotoxicity because they easily germinable and well known from the histological point of view. *R. sativus*, *L. sativa*, *L. sativum*, and *S. lycopersicum* seeds were purchased from the Blumen Group s.r.l. (Emilia Romagna); *L. multiflorum* seeds were purchased from Fratelli Ingegnoli Spa (Milano, Italy); and *P. oleracea* seeds from W. Legutko s.r.l. (Jutrosin, Polland). The seeds were surface sterilized in 95% ethanol for 15 s and sown in petri dishes (Ø = 90 mm) containing three layers of Whatman filter paper impregnated with distilled water (7 mL, control) or the tested solution of the essential oil (7 mL) at different doses. The germination conditions were 20 ± 1 °C with a natural photoperiod. The EOs, in a water–acetone mixture (99.5:0.5), were assayed at the doses of 100, 10, 1, and 0.1 µg/mL. Controls performed with the water–acetone mixture alone showed no appreciable differences in comparison to controls in water alone. Seed germination was observed directly in petri dishes every 24 h. A seed was considered germinated when the protrusion of the root became evident [43]. After 120 h (on the fifth day), the effects on radical elongation were measured in centimeters. Each determination was repeated three times, using petri dishes containing 10 seeds each. Data are expressed as the mean ± SD for both germination and radical elongation.

### 4.7. Statistical Analysis

All experiments were carried out in triplicate and the results are expressed as mean ± standard deviation (*n* = 3). Data of each experiment were statistically analyzed using GraphPad Prism 6.0 software (GraphPad Software Inc., San Diego, CA, USA), followed by comparison of means (one-way ANOVA) using Dunnett’s multiple comparisons test at the significance level of *p* < 0.05.

## 5. Conclusions

The EOs analyzed showed marked and selective phytotoxic properties, inhibiting the radical elongation of *R. sativus*, *L. sativum*, and *L. multiflorum* and the germination of *P. oleracea*. *P. vera* and *Z. officinale* EOs were more phytotoxic than *C. sempervirens* and *C. sativa* EOs; in fact, they were able to inhibit both weeds and food crops, so their use as a mulching material for crops or as a promising chemical herbicide alternative should be evaluated in depth. 

This work opens new research perspectives on plant waste materials, in particular for aromatic plants. In fact, EOs are well known and studied for their antimicrobial and phytotoxic properties. However, they have a rather high cost due essentially to the value of the raw material. In this regard, the use of waste products or spent materials to obtain EOs useful in agriculture as phytotoxic and antimicrobial agents would be desirable. In light of this, further research is needed in order to support this thesis, investigating the phytotoxic activity of these EOs on other weed and food crop species and the mode of action by which EOs exert their allopathic activity. 

Moreover, considering that EOs act and volatilize very quickly, further studies on alternative formulations, such as microencapsulation, are necessary to increase EO efficacy and reduce EO volatilization. 

## Figures and Tables

**Table 1:** Chemical composition of *C. sativa* L. var. *Futura 75* EO.

	Compound Name	Area (%)	KI ^a^	Identification ^b^
1	Heptanal	0.02	217	1,2
2	α-Thujene	0.11	348	1,2
3	α-(+)-Pinene	7.82	401	1,2,3
4	Camphene	0.21	443	1,2,3
5	(1S)-(−)-β-Pinene	3.73	565	1,2,3
6	β-Myrcene	9.32	639	1,2,3
7	α-Phellandrene	0.44	662	1,2
8	δ-3-Carene	0.68	680	1,2,3
9	(+)-4-Carene	0.38	699	1,2
10	*p*-Cymene	0.09	722	1,2
11	D-Limonene	2.92	743	1,2,3
12	*cis*-β-Ocimene	0.54	769	1,2
13	*trans*-β-Ocimene	4.62	810	1,2
14	γ-Terpinene	0.30	827	1,2
15	α-Terpinolen	9.35	919	1,2
16	β-Linalool	0.10	934	1,2
17	Fenchol	0.05	961	1,2
18	L-*trans*-Pinocarveol	0.01	1015	1,2
19	Borneol	0.03	1070	1,2
20	4-Terpineol	0.09	1093	1,2
21	*p*-Cymen-8-ol	0.09	1109	1,2
22	α-Terpineol	0.05	1118	1,2,3
23	*n*-Hexyl butyrate	0.04	1123	1,2
24	(R)-(+)-β-Citronellol	0.02	1184	1,2
25	Bornyl acetate	0.08	1270	1,2,3
26	(+/−)-Lavandulol acetate	t	1278	1,2
27	Ylangene	0.06	1379	1,2
28	Copaene	0.05	1384	1,2
29	(+)-Sativene	0.37	1403	1,2
30	Isocaryophillene	0.46	1419	1,2
31	α-Caryophillene	21.68	1447	1,2
32	β-Gurjunene	0.03	1451	1,2
33	α-bergamotene	3.22	1456	1,2
34	γ-Gurjunene	0.05	1459	1,2
35	β-Caryophillene	9.86	1478	1,2
36	β-Farnesene	0.80	1480	1,2
37	*allo*-Aromadendrene	1.07	1483	1,2
38	α-Selinene	0.02	1485	1,2
39	γ-Cadinene	0.42	1494	1,2
40	β-Himachalene	0.19	1497	1,2
41	γ-Selinene	0.49	1500	1,2
42	α-Guaiene	1.67	1506	1,2
43	δ-Guaiene	2.16	1514	1,2
44	α-Muurolene	0.08	1516	1,2
45	β -Bisabolene	1.23	1526	1,2
46	γ-Muurolene	0.31	1530	1,2
47	(+)-Valencene	0.12	1538	1,2
48	β-Sesquiphellandrene	0.80	1542	1,2
49	β-Panasinsene	1.72	1554	1,2
50	δ-Selinene	1.20	1558	1,2
51	Selina-3,7(11)diene	2.54	1561	1,2
52	Elixene	0.76	1575	1,2
53	β-Maaliene	0.41	1579	1,2
54	*trans*-Nerolidol	0.45	1582	1,2
55	Caryophillene oxide	3.83	1602	1,2
56	Eremophilene	0.09	1618	1,2
57	Caryophylladienol II	0.26	1653	1,2
58	(+)-epi-Bicyclosesquiphellandrene	0.30	1657	1,2
59	α-Cadinol	0.13	1670	1,2
60	(+)-Ledene	0.09	1673	1,2
61	α-Bisabolol	0.13	1697	1,2
62	Guaia-3,9-diene	0.01	1702	1,2
63	Juniper camphor	0.07	1710	1,2
64	Z-9-Pentadecenol	0.02	1742	1,2
65	Hexahydrofarnesyl acetone	0.04	1859	1,2
66	Methyl palmitate	0.02	1940	1,2
67	Biformen	0.08	2005	1,2
68	Dehydroabietan	0.04	2069	1,2
69	Heneicosane	0.02	2114	1,2
70	Phytol	0.06	2128	1,2
71	Cryptopinone	0.03	2178	1,2
72	Dehydroabietal	0.08	2282	1,2
73	Methyl isopimarate	0.02	2310	1,2
74	Tricosane	0.01	2315	1,2
75	Methyl dehydroabietate	0.03	2355	1,2
76	Cannabidiol	1.17	2445	1,2
77	Cannabichromene	0.07	2450	1,2
78	Tetracosane	0.03	2716	1,2
79	Nonacosane	0.06	2916	1,2
	Total	100.00		
	Monoterpene hydrocarbons	40.51		
	Oxygenated monoterpenes	0.52		
	Sesquiterpene hydrocarbons	52.26		
	Oxygenated sesquiterpenes	4.87		
	Cannabinoids	1.24		
	Others	0.60		
	Essential oil yield % (*v*/*w*)	0.2		

^a^ Linear retention index on an HP-5MS column; ^b^ Identification method: 1 = linear retention index; 2 = identification based on the comparison of mass spectra; 3 = Co-injection with standard compounds; t = traces, less than 0.01%.

**Table 2:** Chemical composition of EO from *P. vera* L. var. *Bronte* hull.

	Compound Name	Area (%)	KI ^a^	Identification ^b^
1	Bornylene	0.03	916	1,2
2	Tricyclene	0.72	923	1,2
3	α-Pinene	22.65	935	1,2,3
4	Camphene	3.88	950	1,2,3
5	β-Pinene	1.02	978	1,2,3
6	β-Myrcene	2.43	993	1,2,3
7	2-Carene	1.05	995	1,2,3
8	α-Phellandrene	0.47	1006	1,2
9	δ-3-Carene	7.98	1011	1,2,3
10	α-Terpinene	2.33	1018	1,2,3
11	*p*-Cymene	1.42	1027	1,2
12	D-Limonene	8.50	1031	1,2,3
13	*trans-β*-Ocimene	0.48	1050	1,2
14	*cis-β*-Ocimene	0.35	1056	1,2
15	γ-Terpinene	0.58	1061	1,2
16	4-Carene	32.03	1082	1,2
17	α-Pinene oxide	0.69	1096	1,2
18	Linalool	0.38	1101	1,2,3
19	2-Fenchanol	0.44	1107	1,2
20	1,3,8-*p*-Menthatriene	0.17	1130	1,2
21	Camphor	0.22	1148	1,2
22	Menthone	0.35	1150	1,2
23	Borneol	1.03	1169	1,2
24	*p*-Cymen-8-ol	0.74	1188	1,2
25	α-Terpineol	3.99	1194	1,2,3
26	Myrtenal	0.03	1197	1,2
27	Myrtenol	0.06	1202	1,2
28	α-Methylcynnamaldehyde	0.05	1210	1,2
29	Piperitone	0.53	1250	1,2
30	Nerol	0.34	1232	1,2
31	Bornyl acetate	2.37	1285	1,2,3
32	Nerol acetate	0.18	1365	1,2
33	β-Bisabolene	0.05	1513	1,2
34	γ-Selinene	0.09	1525	1,2
35	δ-Cadinene	0.07	1530	1,2
36	cis-5-Dodecenoic acid	0.13	1568	1,2
37	1,13-Tetradecadiene	1.43	1810	1,2
38	1-Hexadecanol	0.13	1880	1,2
39	Palmitic acid	0.05	1957	1,2
40	1,15-Hexadecadiene	0.43	2549	1,2
	Total	99.87		
	Monoterpene hydrocarbons	86.20		
	Oxygenated monoterpenes	11.37		
	Sesquiterpene hydrocarbons	0.21		
	Oxygenated sesquiterpenes	0.00		
	Others	2.22		
	Essential oil yield % (*v*/*w*)	0.3		

^a^ Linear retention index on an HP-5MS column; ^b^ Identification method: 1 = linear retention index; 2 = identification based on the comparison of mass spectra; 3 = Co-injection with standard compounds.

**Table 3:** Chemical composition of *Z. officinale* L. rhizome EO.

	Compound Name	Area (%)	KI ^a^	Identification ^b^
1	α-Pinene	2.47	861	1,2
2	Santolina triene	6.29	873	1,2
3	β-Pinene	0.45	896	1,2
4	*m*-Mentha-1(7),8-diene	0.32	912	1,2
5	Camphene	1.28	914	1,2
6	α-Phellandrene	0.46	923	1,2
7	*iso*-Sylvestrene	0.06	927	1,2
8	α-Terpinene	0.08	934	1,2
9	Eucalyptol	10.32	949	1,2
10	δ-3-Carene	t	974	1,2
11	(2E,4E)-Heptadienol	t	975	1,2
12	Sylvestrene	t	983	1,2
13	(Z)-β-Ocimene	0.36	997	1,2
14	*m*-Cymenene	t	999	1,2
15	2-Nonanone	0.22	1004	1,2
16	6-Camphenone	0.24	1009	1,2
17	Linalool	0.55	1014	1,2
18	2-Nonanol	0.22	1016	1,2
19	1,3,8-*p*-Menthatriene	t	1019	1,2
20	*endo*-Fenchol	t	1024	1,2
21	trans-*p*-Menth-2-en-1-ol	0.07	1029	1,2
22	*allo*-Ocimene	0.12	1039	1,2
23	2-(1Z)-Propenyl-phenol	t	1041	1,2
24	(3E,6Z)-Nonadienol	t	1044	1,2
25	*iso*-Pulegol	0.14	1054	1,2
26	*neo-iso*-Pulegol	0.71	1062	1,2
27	Borneol	1.52	1074	1,2
28	Terpinen-4-ol	0.13	1083	1,2
29	(E)-Isocitral	0.67	1091	1,2
30	γ-Terpineol	0.54	1098	1,2
31	*trans*-Piperitol	0.06	1100	1,2
32	Citronellol	0.94	1135	1,2
33	Isobornyl formate	4.89	1142	1,2
34	Neral	0.74	1161	1,2
35	2-Pentyl-ciclopent-2-en-1-one	2.07	1172	1,2
36	*p*-Menth-1-en-9-ol	0.15	1182	1,2
37	2-Undecanone	0.60	1193	1,2
38	Undecen-10-en-1-al	0.09	1196	1,2
39	Cyclosativene	0.21	1222	1,2
40	α-Copaene	t	1236	1,2
41	4aα,7α,7aβ-Nepetalactone	0.12	1245	1,2
42	iso-Longifolene	0.39	1248	1,2
43	α-Cubebene	0.69	1260	1,2
44	γ-Elemene	0.27	1275	1,2
45	α-Guaiene	1.17	1278	1,2
46	6,9-Guaiadiene	0.28	1293	1,2
47	*cis*-Muurola-3,5-diene	1.04	1295	1,2
48	*trans*-Muurola-3,5-diene	0.83	1305	1,2
49	α-Humulene	1.92	1312	1,2
50	*allo*-Aromadendrene	2.27	1336	1,2
51	α-Acoradiene	5.27	1360	1,2
52	9-epi-(E)-Caryophyllene	1.56	1369	1,2
53	β-Acoradiene	5.72	1376	1,2
54	α-Zingiberene	15.92	1383	1,2
55	γ-Amorphene	11.55	1384	1,2
56	Viridiflorene	0.06	1395	1,2
57	γ-Patchoulene	8.77	1402	1,2
58	(Z)-γ-Bisabolene	0.37	1407	1,2
59	*cis*-Cadinene-ether	0.22	1411	1,2
60	*trans*-Dauca-4(11),7-diene	0.32	1424	1,2
61	Germacrene B	0.60	1428	1,2
62	*epi*-Cedrol	0.18	1463	1,2
63	1-*epi*-Cubenol	0.47	1486	1,2
64	*allo*-Aromadendrene epoxide	0.38	1495	1,2
65	β-Eudesmol	0.30	1511	1,2
66	α-Cadinol	0.12	1517	1,2
	Total	97.76		
	Monoterpene hydrocarbons	22.72		
	Oxygenated monoterpenes	11.73		
	Sesquiterpene hydrocarbons	60.57		
	Oxygenated sesquiterpenes	1.71		
	Others	3.27		
	Essential oil yield % (*v*/*w*)	0.7		

^a^ Linear retention index on an HP-5MS column; ^b^ Identification method: 1 = linear retention index; 2 = identification based on the comparison of mass spectra; 3 = Co-injection with standard compounds; t = traces, less than 0.01%.

**Table 4:** Chemical composition of EO from *C. sempervirens* L. cones.

	Compound Name	Area (%)	KI ^a^	Identification ^b^
1	α-Pinene	43.25	820	1,2
2	α-Fenchene	0.96	873	1,2
3	Verbenene	0.06	893	1,2
4	Sabinene	3.24	897	1,2
5	β-Pinene	1.95	915	1,2
6	α-Phellandrene	t	924	1,2
7	*p*-Mentha-1(7),8-diene	16.47	932	1,2
8	*iso*-Sylvestrene	0.23	935	1,2
9	*p*-Cimene	t	938	1,2
10	*o*-Cimene	0.44	943	1,2
11	(E)-β-Ocimene	4.49	948	1,2
12	γ-Terpinene	1.37	958	1,2
13	Terpinolene	t	983	1,2
14	*p*-Mentha-2-4(8)-diene	3.76	999	1,2
15	α-Pinene oxide	t	1005	1,2
16	*cis*-Limonene oxide	t	1015	1,2
17	1,3,8-*p*-Menthatriene	t	1020	1,2
18	*trans*-*p*-Mentha-2,8-dien-1-ol	t	1024	1,2
19	allo-Ocimene	2.61	1040	1,2
20	*cis*-*p*-Mentha-2,8-dien-1-ol	t	1046	1,2
21	*cis*-Verbenol	0.17	1048	1,2
22	*trans*-Sabinol	t	1059	1,2
23	(E)-Tagetone	0.06	1073	1,2
24	*neo*-Isopulegol	0.65	1083	1,2
25	*neo*-*iso*-3-Thujanol	0.10	1098	1,2
26	Pentyl cyclohexa-1,3-diene	0.06	1105	1,2
27	(Z)-Isocitral	t	1137	1,2
28	Anisole	0.39	1143	1,2
29	*Neo*-*iso*-Isopulegol	0.64	1180	1,2
30	*cis*-Sabinene hydrate acetate	0.66	1230	1,2
31	α-Cubebene	0.49	1236	1,2
32	α-Terpinyl acetate	2.25	1240	1,2
33	α-Ylangene	0.10	1260	1,2
34	α-Copaene	0.10	1278	1,2
35	Longifolene	0.58	1286	1,2
36	α-Cedrene	3.97	1294	1,2
37	α-Gurjunene	2.01	1305	1,2
38	α-Santalene	1.30	1329	1,2
39	β-Cedrene	0.62	1339	1,2
40	β-Copaene	1.67	1354	1,2
41	Aromadendrene	1.06	1372	1,2
42	γ-Elemene	0.48	1388	1,2
43	Valencene	0.62	1399	1,2
44	Caryophyllene oxide	0.17	1499	1,2
45	Cedrol	0.24	1470	1,2
46	α-Acoradiene	0.12	1477	1,2
47	Himachalol	0.15	1507	1,2
48	α-Cadinol	0.24	1517	1,2
	Total	97.73		
	Monoterpene hydrocarbons	81.34		
	Oxygenated monoterpenes	3.96		
	Sesquiterpene hydrocarbons	13.30		
	Oxygenated sesquiterpenes	0.94		
	Others	0.46		
	Essential oil yield % (*v*/*w*)	0.6		

^a^ Linear retention index on an HP-5MS column; ^b^ Linear retention index on an HP-Innowax column; ^c^ Identification method: 1 = linear retention index; 2 = identification based on the comparison of mass spectra; 3 = Co-injection with standard compounds; t = traces, less than 0.01%.
